# Book Reviews

**Published:** 1814-02

**Authors:** 


					Dr. Ferriar's Medical Histories and Reflections. 145
CRITICAL ANALYSIS
OF RECENT PUBLICATIONS
IN THE
DIFFERENT BRANCHES OF PHYSIC, SURGERY, AND
MEDICAL PHILOSOPHY.
Medical Histories and Reflections; Vol. IV. By John Fer-
uiar, M.D. Senior Physician to the Manchester Infirmary*,
Dispensary, Lunatic Hospital and Asylum. Svo. Loncl.
pp. 117.
]N the first volume of the Medical Histories and Re-
flections, published originally in 1792, Dr. Ferriar com-
municated the results of an extensive series of observations on
the comparative efficacy of different hydragogues and diu-
retics, among which the supertartrate of potass appeared to
him entitled to the first rank. In the second edition of the
same work, in 1810, he repeated his conviction of the virtues
of that remedy; but stated, that he had occasionally found
great assistance from a combination of liquid diuretics, and
that in hydrothorax the symptoms pf effusion were more er-
3 fectuaily
144
Critical Analysis.
fectually relieved by Elaterium, given in small doses, thane
by any other medicine. A case of general dropsy, however,
having occurred, in which other hydragogues afforded no
relief, he had recourse to the elaterium, which acted so suc-
cessfully, as to supersede the operation of tapping, which
liad been previously proposed, aud, in a short time, to re-
move the swellings, and to restore the patient to a comfort-
able state of health. Encouraged by this success, Dr. Ferriar
proceeded to make trial of the elaterium in other cases,
giving it commonly in solution, as in the following
formula:?
ft. Extract. Elaterii, gr. j.
Sp. /Ether. Nitros. gij.
Tr. Sciil.
Oxymel. Colchic. a a gfs.
Syrup. Rhamni, 2*j. Misce.
ft. Solutio.? C'apt. 3j. ex aqua; pauxillo, terquaterve
indies.
Most of the instances, in which it was thus exhibited,
were highly favorable to its efficacy, and our author thinks
that in the above combination he has discovered " a more
certain hydragogue than any which the faculty have hitherto
been accustomed to employconceiving that elaterium
possesses ce a complete power of removing simple effusion,
where no organic disorder exists, and of at least alleviating
the agonies arising from'hydrothorax and ascites, even in
the advanced stage of an incurable disease." We sincerely'
wish that tljis may prove the case; but we think it right to
state, that during the short interval that has elapsed since
the appearance of the volume now before us, we have had
the opportunity of trying the elaterium Vn the manner re-
commended by Dr. Ferriar, in ascites, and in general dropsy;
but from the unpleasant symptoms it occasioned, were soon
obliged to abandon its use, Two of the cases, however, in
which we employed it, were too far advanced to admit of
much hope of recovery from any medicines; and in the
third, the patient took a disgust to it from its first admi-
nistration ; so that our experience cannot by any means he
put in competition with that of Dr. Ferriar; and we shall
therefore suspend our opinion till further trials have enabled
us to appreciate more accurately its powers. In the mean
time, we submit to the consideration of our readers the fol-
lowing remarks of the author.
" I am aware o! die readiness with which practitioners are induced
to exaggerate the powers of a remedy, which has fulfilled their views,
in situations of peculiar anxiety and interest; but I confess that the
nearly uniform result which 1 have experienced from the exhibition
of
Dr. Ferriar's Medical Histories and Reflections. 145
of Elaterium, in hospital as well as in private practice, has impressed
me with the highest opinion of its virtues. During the last three
years, I have made it the leading ingredient in my practice, in this
disorder; and though I have deemed it proper, for the benefit of my
patients, to join active diuretics with it, yet I am persuaded that they
would have proved inadequate to the favorable results of the cases,
without the aid of this e'xcellent hydragogue.
" Indeed I have been convinced, for several years, that modern
practice has been much injured by an affectation of simplicity in pre-
scription, in defiance of the experience of past ages; which has-dege-
nerated in some instances into inertness of composition, and in others
into a thoughtless repetition of a few medicines, applied without
discrimination in most cases. To prescribe, as Crashaw expresses
it, " certain hard words, made into pills," is a wretched prostitution
of a noble art. But this is very different from the powerful combina-
tions which are to be found in the works of the older medical writers.
'The farrago, which was the standing jest of medical men, not many
years ago, must contain unsuspected powers, or it would not have
been employed by such physicians as Sydenham, Willis, or Hoffman.
This appears to me a subject of great interest and curiosity, deserving
the investigation of intelligent observers. I have found the combi-
nation of many liquid diuretics eminently useful; and I have been
pursuing, for some years past, inquiries into the effect of a farrago of
narcotics, from which I flatter myself that beneficial consequences
have resulted, a view of which I may hereafter communicate to
the public.
" It may appear to some persons a fanciful idea, but I have been
led by my observations to suspect that there exists, in the relative
?fleets of medicines, something similar to the harmony of colors and
sounds; and that the impulse requisite to the living powers of the
body, which cannot be produced by a single impression, may be
effected by a concurrence or succession of impressions, in some mea-
sure dependent on eacJj other.
" It appears, from some of the cases which I have mentioned, that
even Elaterium suffers a diminution of its power, from repeated exhi-
bition. In this event, the action of the kidnies may be again ex-
cited, by combining it' with black Hellebore or Gamboge, and by
giving the Syrupus Rhamni, with Oxymel of Colchicum, and a liquid
preparation of Squill, at proper intervals.
" From my experience of the action of Elaterium, it appears to be
particularly exerted in stimulating the absorbent vessels. If this fact
should be confirmed by farther trials, it would lead to an extension
of its employment, in diseases for which at present we can scarcely
be said to possess any remedy. In all cases of preternatural changes
in the growth and organization of parts, in the enlargement and in-
duration of internal glands, in morbid accumulations of animal oil,
and in the destructive process generated by hydatids, we might find
some resource in this active stimulant. But this is advanced simply
as a conjecture; for my experience does not, at present, warrant any
hopes of so flattering a nature."
The second part of the present publication is occupied
?o. ISO, u with
M(3 Critical Analysis.
with the detail of ten cases of Diabetes, in which I)r. Ferriar1
pursued his tonic plan of treatment, as described in the last
edition of the preceding volumes, generally with the effect
of diminishing the quantity of the urine, and its saccharine
quality; and of restoring, at least for a time, the general
health of the patient. On looking over the tables which he
has furnished of the comparative quantities of the ingesta and
egesta in some of the cases, we cannot say that the results
have appeared to us altogether satisfactory; the diabetic
fluid being but slowly diminished, and often rising in quan-
tity above the drink, even towards the termination of the
case. From the* recent experience of Dr. Warren, Ave are
inclined to think, that if Dr. Ferriar had administered the
Opium more freely with the Cinchona and Uva Ursi, the
effects would have been more marked. It is certainly, how-
ever, much in favor of his plan, that of the thirteen cases of
which he has preserved minutes, ten have been cured, two
much relieved. One of the patients, Winterbottom, whose
case was given in the second edition of the Medical Histories,
lias now remained four years entirely free from diabetes.
To the history of these cases are subjoined some remarks
on the theory of Diabetes, in which the author has " ven-
tured," as he himself Acknowledges, " on the dangerous
ground of hypothesis." Being prepossessed in favor of no
particular theory of this puzzling disease, we may reckon
ourselves impartial judges of that now before us; and dis-
posed, as we are, to treat with respect every thing which
comes from the philosophic mind of Dr. Ferriar, we have
no hesitation in affirming, that, by referring the symptoms
of diabetes-to a diseased action of the extreme vessels, in
consequence of which " they form a substance which cannot
be applied to renew the waste of th.e system, instead of sup-
plying proper nutritious matter," we should explain little
or nothing; as the cause of this diseased, action would still
remain to be ascertained. In place of such lvypothetical
reasoning, which, in our present state of knowledge, is evi-
dently premature, we should have more gladly received a
detail of experiments on the state of the different secretions
and excretions in diabetic patients, particularly those of the
skin, lungs, and alimentary canal, which have been hitherto
not at all, or at least very imperfectly, examined. Such ex-
periments we know that Dr. Ferriar has ample opportunities
of instituting; and he will agree with us, that it is only by
such a mode of proceeding that we can ever legitimately
arrive at the right explanation of the disease.
The volume concludes with a case of Scirrhus of the
Pylorus, which is chiefly remarkable as exhibiting a strongly
marked example of this incurable complaint.
i Medical
147
Medical Transactions, published by the College of Phy-
sicians in London. Vol. IV. pp. 415.?Longman and
Co. 18 J 3.
(Continuedfrom p. 79 )
In resuming our analysis of this valuable work, we shall
pursue our plan of extracting as much of those papers
which afford the most interest, as the limits of our Journal
will admit of.
VI. Of a peculiar Affection of the Eyes, with Observations.
By W. Heberden, M.D. F.R.S.
"In the month of July, 180(5, a lady of advanced age
went from London to the eastern coast of Kent, where she
lodged in a house, looking immediately upon the sea, and of
course very much exposed to the glare of the morning sun.
The curtains of the bed in which she slept, and also of the
windows, were of white linen, which made her apartment
very light. When she had been there about ten days, she
observed one evening, at the time of sun-set, that first the
fringes of the clouds appeared red, and soon after the same
color was diffused over all the objects round her. It was
particularly conspicuous when she regarded any thing white,
as a sheet of paper, a pack of cards, or a lady's gown. This
lasted the whole night. The next morning her sight was
perfectly restored. But as the evening advanced, the same
appearance came on again ; and it continued to do so regu-
larly every evening, as long as she remained at that place,
which was three weeks from the commencement of her com-
plaint ; the natural vision always returning in the morning.
Six days after she had left the coast, I saw her in London,
still subject to the same affection ; and she has since informed
ioe that it persevered a fortnight longer, and then of its own
accord ceased suddenly and entirely. While it was upon
her, the sight seemed to be no otherwise impaired than by
the degree of indistinctness necessarily produced by this
unnatural color, which overspread all her view.
" There seems great reaion to suppose that this lady's
complaint was brought on by her being exposed to an unu-
sual glare of light. Previous to this siie had been harassed
by a cold and cough, which had hung upon her longer than
usual, but she had suffered no disorder in her eyes."
VII. Some Observations on the Scurvy. By W. ITebeii-,
den, M.D. F.R.S.
Although this paper displays much research and minute
observation, we shall not detain our readers with an account
qf it, as the disease to which it relates is now rarely seen in
this country; and practitioners in the navy would probably
u 2 disdaiq
148 Transactions of the College of Physicians.
disdain the remarks of a landsman, however excellent, upon
a disease of which he had only met with a few instances
during many 3?ears practice in an hospital.
VIII. Observations on the internal use of Nitrate of Silver, in
certain convulsive Affections. By Richard Powell, M.D.
This paper commences with a sort of historical account of
the use of argentum nitratum as a medicine, which, of course,
can contain nothing new. The author has given it in the
form of a pill to the extent of fifteen grains for a dose. He
has adduced some cases which will illustrate its effect, as
well as the nature of the diseases in which benefit may be
expected from its use.
" E. H., a girl of eleven years of age, had a permanent
contraction of the flexor muscles of the right arm, by which
the hand was drawn to the shoulder. This local affection
?was not attended by any general derangement of the consti-
tution, and she was admitted into the hospital, December
3d, 1801. Slight electric shocks were first passed along the
course of the contracted muscles, by which they were spee-
dily relaxed: but a similar affection of the left leg immedi-
ately succeeded. This also yielded to the same application,
when the right leg became in turn similarly diseased ; and
on passing the shocks along it in like manner, an affcction
of the head with general c'onvulsion and stupor came on,
"which lasted for about ten minutes ; and on her recovery
therefrom, the leg alone remained contracted. Subsequent
repetitions of the electricity produced the same series of
effects more than once ; but as the hazardous affection of
the brain occurred in every trial, and nothing but a changc
of situation was gained by it, the practice was not persevered
in. She took without effect sulphate of zinc, precipitated
green oxyd of iron, with myrrh, camphor, opium, oil of
amber, and animal oil, and used the warm bath and other-
local applications. Nitrate of silver was lastly given in pills^
first of half a grain , then of a grain*, three, four, and six times
in the day, and from this alone did she receive any benefit.
In about three weeks she was perfectly free from contrac-
tion ; but on quitting the hospital, and discontinuing the
medicine, the disease again recurred, and again yielded to
the same remedy, which was then however longer continued :
and when I last saw her, she had remained well for three
months. The medicine produced no immediate or sensible,
effect, but her power of extending the arm gradually in-
creased.
u R. N., aged eleven years, a boy of uncommon mental
powers, but of a vicious uncontrolable disposition, was af-
fected
Br. Powell on the Use of Nitrate of Silver in Convulsions. 149
fected with chorea, and had long been under the care of
my friend Mr. Anderson. He had taken antispasmodics and
metallic tonics, and latterly also arsenic, without effect;
when Mr. Anderson, with this information, wished me to try
what could be done for him under the restraint and super-
intendance of the hospital. I admitted him in February 180(5,
in the hope that purgative medicines would succeed, and
gave him ten grains of the pulvis scammonii cum camelolane,
which I repeated every third day with a considerable pur-
gative effect, which was kept up during the intervals by a
solution of a drachm and a half of magnesia vitriolata every
six hours. He was thus'actively purged ; but after ten days*
trial, all the symptoms were aggravated, and I did not think
farther perseverance justifiable. Metallic tonics had been
judiciously given : I had next therefore recourse to digitalis,
in doses of half a grain every six hours, and afterwards to
half a grain of opium, at the same intervals. The coid bath
was also tried ; but every immersion increased the convul-
sive motions, and the boy grew rapidly worse, until at last
the convulsions had become constant even during sleep, and
if he was noticed, he could with difficulty be confined to
his bed. I directed the argentum nitratum, which had not
hitherto been given, in doses of a grain every four hours,
and within a week some ground appeared to have been
gained. It was therefore increased by gradual additions of
half a grain, urttil he took three grains every four hours,
from which quantity the beneficial effect was so clear that I
increased it no farther, and in six weeks from the commence-
ment of its use, he wras dismissed cured. I find from subse-
quent inquiry, that there was no return of the complaint.
The medicine was given in solution, and the last increase of
it in this form produced slight sickness the first day it was
taken, but no other sensible effect followed, although the
_ quantity amounted to eighteen grains in twenty-four hours.
" W. B., a boy of fourteen years of age, had previously
been my out-patient with this complaint, and had received
110 benefit from the usual remedies or from active purgatives.
I therefore admitted him into the house on August 14, 1800,
for the purpose of a regulated trial of argentum nitratum.
I began with halfa.grain every four hours, and increased
the dose gradually Until September 16, when he took four
grains in solution every four hours. Amendment was visible
within a fortnight; but the last increase of dose having oc-
casioned some sickness and pain at the stomach, 1 diminished
it to three grains, in which he persevered without inconve-
nience. I have no statement respecting any effect upon his
bowels^ but he regained in the house till the end of Octo-
ber,
150
Transactions of the College of Physicians.
ber, 1800, 011 account of an injury to his knee which re-
, quired the care of the surgeon, and he continued the use
of the medicine the whole of that time.
" S. S., aged fifteen, a girl of irritable mind, and who
had not hitherto menstruated, had been attacked three weeks
before her admission with convulsions 011 the left side only,
which she believed to have originated from sudden fright.
The disease was growing worse, and on Januarys, 1807,
?was very violent. I gave her an active dose of calomel and
jalap, which procured four purgative motions without any
particular appearance. She then began the use of argent urn.
nitratum in solution, first in the dose of one grain every four
hours, and on February 4, four grains at the same intervals.
Until the last increase of dose she had only one motion daily,
after it she had two, for two successive days, but the con-
vulsions had gradually abated ; and on February 9, I con-
sidered her as free from complaint. She remained, however,
under the use of the same medicine for a fortnight longer.'1
IX. On the Mortality cf London. By William Heber-
den, M.D. F.R.S.
From this paper it appears, that during the last fifty years
the healthiness of this metropolis has materially improved.
Since the year 1800, the christenings exceed the burials in
the proportion of 13 to 12 ; and by accurate calculations,
London now ranks among the most health}' of large towns.
This diminished mortality is attributed by the learned author
to the widening of the streets, the removal of nuisances, the
opening of confined quarters, the erection of public squares,
the construction of better drains, and the universal diffusion
of water pipes.
X. Some Observations on Phthisis Pulmonalis. By Edward
Roberts, M.D. F.R.S.
XI. Observations upon the comparative Prevalence of Insanity,
at different Periods. By Richard Powell, M.D.
The subject of this paper is important, and the author,
from his official situation as secretary to the commissioners
for licensing and regulating houses for the reception of luna-
tics, is possessed of ample means for pursuing the inquiry.
The length of the article, however, precludes us from giving
the whole of it, and it cannot be abridged with advantage.
XII. Case of Sup erf (station, communicated by William
George Maton, M.D. F.R.S.
<c Mrs. T. (an Italian lady) was delivered of two male
.children, at Palermo, on the 2d of June ISOG; one of these
children
Dr. Mat on on a Case of Superfoetation. 131
children lived about two years, and the other three years,
and are supposed to have died more from want of care than
< from any other cause.
" On the 12th of November 1807, she had another male
child, who was brought forth under circumstances very dis-
tressing to the parents, being dropt on a bundle of straw, at
midnight, in an uninhabited room. Though this infant had
every appearance of health at the time of his birth, he lived
about nine days only.
" But the occurrence which forms the more immediate
object of this paper is now to be related. On the 2d of
February 1808, (not quite three calendar months from the
preceding accouchement.,) Mrs. T. was delivered of another
male infant, completely formed, and apparently in perfect
health. He was sent away to be nursed, but the nurse'*
milk being deficient, he was removed soon afterwards to
another foster-mother. When about three months old, how-
ever, he fell a victim to the measles, and died. From No-
vember 1807? to February 1S0S, Mrs. T. had not left Pa-
lermo, except on short excursions in her own carriage ; and
Mr. T. the lady's husband, (who is in the commissariat d?-
partment of the British army,) has been constantly with his
wife since the year i 805. The prolific character of Mrs. T.'s
constitution is, perhaps, almost unprecedented ; for, on the
23d of November 1808, she again had twins (males) who
are both alive, and in good health, at Palermo. On the 9th
of June ] 809, Mrs. T. miscarried, on-board of the ship
which brought her to England, and which was preparing to
engage some cruisers. She is pregnant at this period.
" I learn that Mrs. T. is of so robust a habit, that she pays
scarcely any attention to her health. She was carried from
the room where she was delivered of the child, in February
1803, to her own apartment, in the arms of her husband,
and in two or three days afterwards walked about the house.
" In a certificate which Mr. T. was so obliging as to send
tome, he solemnly pledges his word to the correctness of the
above statement, except so far as relates to the precise time -
which the several children lived, and which may have been
a few days longer, or shorter, than the periods which have
been mentioned. The circumstances were first made known
to me by Mr. James Thomson, surgeon, of Tavistock-place,
Russell-square, who had the goodness to acquaint his friend,
the husband of the lady, with my wish to communicate them
to the Royal College of Physicians. That there might be
no mistake, the latter gentleman then committed them to
paper, attested by his signature, which he politely put into
my possession.
" I shall
152 Transactions of the College of Physicians.
" I shall not attempt to connect the case which I have
described, with any opinions respecting the process of gene-
ration, but content myself with opposing it to the scepticism
of some modern physiologists, who appear to me to discoun-
tenance all belief in the occurrence of superfcetation, more
from partiality to a favorite hypothesis, than from any clear
notions, as yet established, of the mode in which impregna-
tion takes place.
" The case of superfcetation related by Harvey [de par lit
Exercit. p. 547}, with regard to the time that intervened
between the two births, is the same as Mrs. T.'s."
XIII. /I Case of Tetanus arising from a Wound, in which the
Affusion of cold Water was successfully employed. By George
Gilbert Currey, M.D.
This is a valuable case, and clearly demonstrates the
power of cold water in subduing a violent and dangerous
disease.
XIV. Sequel to the Paper on Tetanus. By
J. Latham, M.D. F.R.S.
A man intent on serving the public, will rather incur the
charge of doing too much than too little : our high respect
for the president of so learned a body as the College of Phy-
sicians, compels us to wish that he had not published this
paper. The object of it is to establish an unnatural analogy
between Hydrophobia and Tetanus, and to recommend " the
Eau Medicinale d'Husson" in the former malady, because it
has been said to diminish painful and inordinate action in Gout.
.But if this precious nostrum cannot readily be obtained, Dr.
Latham thinks that the " Vinum Veratri," as prepared at
Apothecaries' Hall, " would probably be no less efficacious!"
XV. Case by Mr. Blagdon, Surgeon, at Petworth. Com?
- municated by W. Saunders, M.D. F.R.S.
This is the case of an old lady, who, after hepatitis, had
an abscess in the liver, and afterwards discharged some sail-
stones, the appearance of which is described in a colored
plate.
XVI. Two Cases of Diabetes Mellitus, treated with Opium.
By Pelham Warren, M.D. F.R.S.
These are two clear and well marked cases of Diabetes, in
which animal diet and opium evidently had a beneficial ten-
dency in effecting the cure. We have not space sufficient -
to insert these cases, but the following Tables will show the
doses of the medicines employed, and the state of the urine
during their use. * " 1
/
" CASE
Dr. Warren on Opium in Diabetes Mellitus. 253
" CASE OF JOHN TRANTER.
Table of the progressive state of the Urine and Drink, measured by
Pints, and of the Opium administered.
1812.
I Feb.121
111
19
25 9
| Mar. 2
67
10
13
15
16
20 7
24
27
April 3 7
10 7
13
17 7
18
20
24 6
2 7
May i|7
81?
15 7
2217
25
26
29|
| rnne 5
5?6
tiiiantities foi
No. of Days.
Greatest and least
quantities per dien
Urine.|!)rink
11
lC'^j39j4^-
4
52
49,
34
29
24
H
54
25 3yj
25 3d
18
>8;6
6f
3891
'o2 8f
G3
kjisf
39
35
29
34-14
34
64
Urine,
10
10
10
12i|
91104
7i
9 7
10
541
H
Drink.
10
44
5?
89
894
84
H
3 4
4 4
Opium and other
Medicines.
Pulv.Ipec.G.9j. o.n
Cue Cr. ad Jvj. Em
lytta; iurnhis & pulv
Ipec. C. 9j bis die.
Pulv. Ipec. C. $ss I,;
_9j_
9iiss. ?J
3j -
5j seinel
9'j t>i
Be Kino 9j ()pii
gr. iv
gr. v
gr. vj
Omittantur Kino
& Opium donee
alvus soluta fu-
erit
R Kino 9J Opii
gr. viss
Haust. emeticus.
[Iaust. purg. hodk
& eras.
[Omittantur Opium &
Kino. Haust. purg.
Resumantur Opium &
Kino.
Magnes. 9i cum
sing. dos. Kino &
Opium.
[Omittantur Opium &
Kino. P. in usu
Magnesia. Ilaust
purgans.
Magn. Sulp. Jvj'.
Jpii gr. iij bis die.
State ol|
Urine.
>%
CO
?Z I
>2
l CO
Np. 180.
X
TABLlii
154 Transactions of the College of Physicians,
" TABLE II.?CASE OF THOMAS ANDREWES.
Table of the progressive stale of the Urine and Drink, measured by
Pints, and of the Opium administered.
1812.
jQuantitieifot
No. of Days-
|June22
26
29
jJuly 3
C
10
13
17
20
24]
27j
31
I Aug.
?
1(1
1'
17
24
3]
jSept. f
7
14
19|
21
2i
lOct. 5|
1'.
19]
20
9?
UrinelDrink
4
52
w>
7|8
Greater and least
[quantities per dieml
Urine. | Drink.
H
Opinrn and other
Medicines.
Op.i gr. ij o. n.
bis di<
gr. nss
gr- iij
gr. iv
gr.ivss
gr. v
Nihi
V. S. ad ?viij,
[Opii gr. iij bis dif
? Kr? 'v
Empl. lyttse
Epigastrio
5 viij Haust.
cetacei cum
oxytn. scillce
. 5' ter ? -
[Omitt. Haust.
cetacei, &c.
gr. v
? quater ?
bis
ft Pil. e micfi panif
Opii gr. v ter die
quater dif
State ol
Urine.
"55 %
1 ^
H
S3
Diet.
. W)
r>
-a
?
)<
*?
1st
}
6!
?a sft
Hi
i
CASE
Dr. Warren on Opium in Diabetes Melliius. 155
CASE OF THOMAS ANDREWES CONTINUED.
1812.
|Nov. 2 7
9 7
11
13
16 7
23 7
30 7
I Dec. 2|
7 7
9
14
18
21
28 7
|Jan. 4 7
6
11
jQuantities for]
No. of Day
Urine iDrink
33
3114
133
2513
28 4
28 4
i*4
28 4
36 4i
13515
26
[32
31
30|
[33
30|4
4*5
4 4
[324^5
132,
Greater and least
[quantities per diem
Urine. I Drink.
3?|4
44
S
4 U
2 3
2*3*
Opium and other
Medicines.
Opii gr. vj
gr-viij
gr. ix ?
gr. x
V. S. ad
iv"J
ter ?
bis ?
?gr.vmj.
? Sr- x ?
? gr. iiss ?
? gr. iv ?
? gr. v -
gr- vj
Rc Pulv. Cinchon ?
9j Pulv. Arom.
gr. v ?
Oinittr. ()piiiii
Opii gr. vi seme1
[State of |
Urine.
The patient Andrewes soon afterwards died of a pulmonary
complaint, which had affected him during the course of the
diabetic disorders, and afforded Dr. Warren the opportunity
of observing the appearances after death, which are thus
detailed in an appendix.
" The body was exaqiined twenty-four hours after death,
when the following appearances were observed. The heart
was small and flaccid, the pericardinm did not contain any
water. The right lung looked healthy externally and was
free from adhesions, but, on cutting into its substance," was
found to contain tubercles in an incipient state. The left
lung adhered so strongly to the pleura costalis that it was
with difficulty detached. On being removed from the body,
the whole substance of it appeared to be diseased, and di-
vided into cells of different dimensions, containing matter.
The cavity of the chest did not contain any water. The
liver was in a natural state. The spleen was sound, and of
x 2 its
156
Transactions of the College of Physicians.
its usual size. The blood-vessels which pass from the spleen
to the liver were of the usual dimensions. A portion of the
greater curvature of the stomach, immediately below the
entrance of the oesophagus, exhibited some patches of super-
ficial inflammation, and at the small end of the stomach ex-
tending a little way into the pylorus, there was a similar
appearance. The intermediate portion of the stomach was
perfectly natural. Upon various parts of the cellular mem-
brane of the abdomen, a gelatinous substance was thrown
out, resembling the jelly which is sometimes found between
the membranes of the brain.
" The following description of the state of the kidneys
was drawn up by Mr. Brodiei the assistant surgeon of St.
George's Hospital, by whom their structure was minutely
examined.
" On examining the kidneys, with reference to the us^ial
appearance of these organs in a healthy state, both of them
were found to be of an unusually firm and grisly texture.
The cellular membrane surrounding the pelvis and infundi-
bula was loaded with serum, and portions of lymph were
diffused through it, bearing the appearance of jelly, and re-
sembling that which had been observed in various parts oi
the cellular membrane of the abdomen. In order to ascer-
tain the state of the blood-vessels, one of the kidneys was
injected with size and vermiilion previously to examination,
the other was examined in its natural state. On cutting
into the substaiice of the injected kidney, the cortical part
appeared unusually red, and the cryptae were seen more
"numerous, Urger, and more distinct, than I had ever ob-
served them, even under circumstances of the most fortunate
injection. The blood-vessels of the cortical substance of the
kidney which had not been injected, were unusually turgid.
The ureters, the emulgent artery and vein were of the na-
tural dimensions. The renal capsules were firmer and harder
in their texture than natural, and seemed to partake of the
grisly structure of the kidneys ; in other respects they were
ljot diseased. The bladder was sound."
XVII. Case of Inflammation, and subsequent Mortification, of
" the Adipose Membrane surrounding both Kidneys; with the
Appearances on Dissection. By Thomas Turner, M.D.
" A married lady, in the thirtieth year of her age, rather
under the middle stature, and very corpulent, on Saturday,
the 21st ol June, rocie 011 horseback for several hours,
By which she was very much heated and fatigued. She sat,
for some time afterwards, in a room with the windows and
4oor open, but was not sensible of any inconvenience from it.
Dr. Turner on a Case of Inflammation. 157
She passed the rest of the clay as usual, and at supper eat
heartily of lobster, although it generally disagreed with her
stomach, and made her ill. On retiring to her bed-room,
she felt sick, and vomited : she also complained of much
pain in her bowels. These symptoms were attributed to her
having eaten the lobster. An emetic was administered by
the advice of the family apothecary, and on the following
morning a purgative draught, which, not operating as soon
as might be expected, was repeated, and then produced
plentiful evacuations from the bowels. In the evening, she
said she felt quite relieved, and had no complaint to make,
except that her hands and feet were colder than usual. She
was advised to go early to bed, and to takesomething warm:
no doubt was entertained that she would be quite well again
on the next day. In the .night, however, she experienced
considerable pain in her back and loins, which she thought
was greatly relieved by having them rubbed for some time.
She became, however, very restless, and about live o'clock
on Monday morning, her restlessness and anxiety had in-
creased to such a degree, that her husband began to be
alarmed. He sent accordingly for the apothecary, who came
in about an hour afterwards. By this time, her extremities
had become quite cold, and no pulsation could be perceived
at the wrist. On his expressing apprehension of immediate
danger I was sent to; but as the patient lived at some dis-
tance from London, I did not see her till between one and
two o'clock in the afternoon. I then learned, that she had
taken some hot brandy and water, with a few drops of tinc-
ture of opium, which appeared to have revived her, so that
her friends thought her better than she had been in the
morning. Her anxiety, however, was extreme, and her
breathing occasionally very much oppressed : her feet, legs,
hands, and arms, were quite cold ; not the slightest pulsation
could be distinguished in the radial artery; her countenance
was livid, and her features drawn up; her mental faculties,
however, were unimpaired. She said she was quite free
from pain : she complained of no uneasiness when I pressed
her abdomen with my hand, neither could I perceive any
unusual tension or hardness. She assured me she had expe-
rienced neither pain nor difliculty in making water, neither
was she aware of its having been less copious than common.
She gradually sunk till about four o'clock, when she expired.
" I obtained leave to examine the body, two days after
ficr death, and the following appearances were observed in
the dissection.
" Kather more than the usu^l quantity of fluid was found
in
138 Transactions of the College of Physicians.
in the cavity of the thorax: all the viscera contained in it
were perfectly sound and natural.
" In the abdomen, slight marks of inflammation were per-
ceived on the peritonaeal coat of the stomach, on that part
of its greater curvature which adjoins the spleen and arch of
the colon.
" The part of the colon which lies immediately over the
left kidney was much inflamed.
The liver was in a perfectly natural state, except that
it had one very small tubercle in the anterior part of its
right lpbe.
" The gall-bladder contained five rather large biliary
poncretions.
u The pancreas was inflamed through its whole substance,
pnd a considerable portion of its left extremity was in a stat^
of mortification.
" The whole of the adipose substance surrounding both
Vidneys, was in a gangrenous state, exhibiting a large mass
of black pulpy matter. The capsules of both kidneys were
inflamed, and that of the right kidney partly mortified:
slight traces of inflammation were also found in the internal
Structure of both kidneys.
ii All the other viscera were iq a natural state.
" We may remark, that, through the whole course of this
disease, the urine was secreted in proper quantity and ap-
pearance ; that there was no pain felt in the ureters, nor any
irritation in the urinary bladder; that the nausea, and paii*
in the bowels, were speedily relieved by the medicines that
were given for that purpose ; a.nd, moreover, that the symp-
toms of general inflammation must, have been very slight, as
they eluded the observation of the very respectable medical
practitioner, who visited the patient frequently, during the
progress of the complaint. We may therefore fairly infer,
that the inflammation did not commence in the kidneys j
neither have we any reason to suppose that it originated in
the pancreas or colon : on the contrary, the whole of the
adipose membrane surrounding the kidneys, i which, in this,
lady, was loaded with a much larger quantity of fat than is.
usually met with,' being fpund in a gangrenous state, there
can be little doubt that this was the part first attacked with
inflammation; that mortification rapidly ensued, and that
then the kidneys, pancreas., and colon, were involved in the
disease, from their continuity to this mass of sphacelated tat.
" Long continued riding on horseback, is mentioned as
qne of the exciting causes of nephritis, by most writers on
the subject; and was most probably the exciting cause of
this disease^ which, in the view I have taken, of it, has not
hitherto,
Dr. Baillie on Pulsation of the Aortal 159
hitherto been noticed by any medical author whom I have
consulted. The ambiguous nature of its symptoms, its rapid
progress, and fatal termination, will entitle it to be ranked
among the most formidable of the internal inflammations."
XVIII. Of Head-achs, which arise from a defective Action
of the digestive Organs. By Pelham Warren, M. D.
F. R. S.
This paper, occupying nearly forty pages, displays much
knowledge upon the subject of which it treats. The symp-
toms are well described and accurately discriminated, and
the treatment recommended is judicious, and doubtless the
result of considerable experience.
XIX. Upon a strong Pulsation of the Aortay in the Epi-
gastric Region. By Matthew Baillie, M.D. F.R.S.
" It has frequently occurred to me to have been consulted,
within the last fifteen years, respecting a pulsation which is
distinctly to be felt in the epigastric region. Wh<Jn the
patient first discovers this pulsation, he is generally greatly
alarmed at it; and has seldom found much comfort from the
opinion which has been given him by his medical attendant
concerning its nature. From a good deal of experience
upon this subject, I am enabled to say, that the increased
pulsation of the aorta in the epigastric region very rarely
depends upon any disease of the aorta itself, or of its large
branches in that place ; and that this occurrence is almost
constantly of very little importance. In the whole course
of my medical experience, I recollect but one instance in
which this pulsation depended upon an aneurismal swelling
of the artery.
" This symptom, or complaint, is more apt to take place
at the middle period of life than at any other; but I have
known one or two instances of it in persons about the age of
thirty. It occurs both in men and women, but more com-
monly in the former than in the latter* In one individual
the pulsation is much more strongly marked than in ano-
ther; and in the same individual it varies a good , deal in its
strength at different times. In some instances the pulsation
is more strongly to be felt in the evening than in the fore-
noon. It is generally more" distinctly to be felt when the
patient is in the horizontal posture ; and sometimes the pul-
sation is so strong as to be visible to the eye, even at some
distance, when the surface of the epigastric region is ex-
posed to view. In some instances, the boundary of the
artery, while it pulsates, can be very distinctly felt, and it
may even occasionally be traced nearly as low as the navel.
I do not recollect that there is any peculiarity in the pulse of
persons--
160 Transactions of the College of Physicians.
persons affected with this complaint. It is commonly neither
intermittent, nor remarkable either for frequency, strength,
or weakness. I have had two opportunities of examining
the state of the arteries in the epigastric region, after death,
in persons who had this pulsation very strongly marked, and
who*had died from other diseases. The one person died
from an ulcer in the stomach; and the other from a very
long typhus fever, where the stomach was sound. In both
cases the aorta itself, the branches of the cceliac artery, and
the superior mesenteric artery, were perfectly free from all
appearance of diseased structure. The arterial system, in
these two cases, was not more extensively examined; but
there was no reason to believe that any part of it was
diseased.
<{ It is, perhaps, difficult to ascertain, in many instances,
the causes of this increased pulsation of the aorta in the
epigastric region; but in most cases it will be found to be
connected with an imperfect digestion, and an irritable con-
stitution. When it has once taken place, it seldom, I be-
lieve, subsides entirely, although it will vary in its degree
at different times. It produces, however, not much incon-
venience, more especially where the mind has ceased being
anxious about it; and a person may continue to live with
this symptom, or complaint, just as long as if it did not exist.
Some years ago I was consulted by an old man concerning
a slight paralytic affection ; and when the conversation re-
specting that part of his complaints was finished, he spoke
to me about a pulsation in the epigastric region, it was
most distinctly to be felt; and he told me, that on account
of this pulsation he had consulted Sir Caisar Hawkins, Mr.
Bromfield, and Dr. Hunter, about twenty-five years before
the time of his consulting me. Sir Cajsar Hawkins and
Mr. Bromfield, he said, told him that it was an aneurism of
the aorta; and Dr. Hunter told him that he did not know
what it was. Dr. Hunter saw enough of peculiarity in this
case to make him doubt, but not enough to enable him to
decide that there was no disease in the aorta.
" This case shows that a person may continue to live for
many years with a strong pulsation of the aorta in the epi-
gastric region ; and, from the appearance of the patient,
his general health seemed to have been very little impaired
by it.
lf It would be a matter of considerable importance, if we
could at all times certainly distinguish between the pulsation
of the aorta about which we are now treating, and that
which is occasioned by an aneurism of the aorta in the epi-
gastric region. This, however, will in some instances be
d hardly
Dr. Latham on Angina Pectoris. lGl
liardly possible, more especially in the early periods of aneu-
rism. When the boundaries of the artery can be felt dis-
tinctly, and the artery can be ascertained to be of the usual
size, it is clear that, notwithstanding the force of the pulsa-
tion, the disease is not aneurism. When a round circum-
scribed tumor pulsates against the fingers applied to the epi-
gastric region, there can then be little doubt that the disease
is aneurism either of the aorta or of the coeliac artery.
When the pulsation has continued for several years without
the health being materially impaired, even if the boundaries
of the artery should not be distinctly felt, yet there is the
strongest reason to believe that the pulsation of the artery
does not depend upon an aneurismal swelling in it.
" I am not acquainted with any means of curing this
sj^mptom, or complaint. Whatever improves the digestion,
and renders the constitution less irritable, will be of use in
mitigating the increased pulsation ; and, above all other cir-
cumstances, it is useful to remove the patient's anxiety on
the subject, when it can fairly be done.
" I liave thought it of some importance to communicate,
through the Transactions of the College, my experience re-
specting this complaint, as it may tend, in many instances,
to relieve the anxiety of the patient, and even sometimes of
his medical attendant. It may likewise, in time, lead to a
successful treatment of the disease, with which the increased
pulsation of the aorta in the epigastric region is connected."
XX. Observations on certain Symptoms, usually, but not.
always, denoting Angina Pectoris. By J. Latham, M.D.
F.lt.S.
In this paper we have some valuable practical observations*
proving that enlargement and disease of the liver, occa-
sionally give rise to symptoms that have been mistaken for
those of angina pectoris, and other affections of the thoracic
viscera, even by very eminent physicians. Dr. Latham has
related two cases in support of his remarks; and as they are
curious and not long, we shall insert them.
" The first was that of a clergyman of the University of
Oxford, and Fellow of Merton College, who had frequently
labored under angina pectoris, and had as often been re-
lieved : the symptoms always returned after short intervals,
and the common diuretic and purgative plans always re- ?'
moved them. In the absence of Dr. Pitcairn, who was then
at Lisbon for the recovery of his health, I was occasionally
employed by the family of this gentleman, and he himself
also became my patient. Although he was speedily relieved,
the symptoms, as usual, soon returned ; and thence con-
no. 180. V eluding-,
362 Transactions of the College of Physicians.
eluding, that as the symptoms were not constant, there could
not be permanent mischief connected with diseased organi-
zation within the thorax, I very minutely examined the
abdomen and its several secretions; and having good reason
to believe the liver to be in fault, I pushed on the mercurial
plan so as to produce a slight ptyalism. The unfavorable
circumstances, as usual, soon disappeared} and after enjoy-
ing more ease and comfort for a much longer interval than
was common with him, I gave him permission to remove
from London to his brother's residence in the country, and
took my leave of him. On the following morning, as he
was preparing for his journey, and bustling a little that he
might not keep the carriage waiting, he suddenly fell down
and expired.
" The body was examined ; and on meeting Dr. Pitcairn,
1 put the question as to what he conceived might have been
the result of the examination. He replied, 4 You would find
organic disease either of the heart or of the larger vessels
certainly.' His surprise was great when I informed him that
there was no disease discoverable throughout the body, ex-
cept an enlargement of the liver, which was thought to be
harder than natural, and which appeared to be pushing
against the diaphragm, so as to form as it Avere a sort of
recess within it, and in such a manner as to diminish the
area of the lungs, and consequently impede the thoracic
circulation.
" Another case was that of a merchant in the city, whose
pulse, though always remarkably slow, was never otherwise
irregular; for during my attendance upon him, which was
occasionally during ten or twelve years, I never discovered
any intermission. His pulse, when he considered himself
well, seldom exceeded 32 beats in a minute; but in his pa-
roxysms of disease, if the application of such a term is allow-
able, the number was usually 22, and once 1 counted only
4 7 strokes. The pulsations might be considered as so many-
heavy undulations, affording, as it were, a chasm in space
as well as time between each ; and during which interval the
muscles of the abdomen and thorax were.so visibly affected
as to remain in a state of tremulous or vibratory motion,
which gradually diminished until the next wave or swell was
succeeded by another similar vibration. I once attended
him through a regular fever, when his pulse was not more
than 60, notwithstanding the disease ran on for at least a
fortnight with hot and dry skin, white and furred and parched
tongue, and occasional delirium. This gentleman was con-
sidered by almost every physician who saw him as laboring
under organic mischief in the thorax; and some thought that
Sir IT. Halford on the Climacteric Disease. ]&>
the brain was the primary seat of the disease, because, as it
should seem, the slowness of the stream sometimes produced
full isess and consequent head-ach. The mischief, however,
appeared to be almost entirely in the liver; for upon a
gentle salivation, long continued, he attained a state of
comfort comparatively great, and for many months after-
wards was almost free from disease. Cheltenham and Bath
were successively resorted to for the benefit of the waters;
but the enlargement of the liver, with its constant pressure
against the diaphragm as the primary cause, had vn tlfis, as
in the before-mentioned case, produced all the other se-
condary effects, and so disturbed the functions of the lungs
and the circulation through them, that one day, when in
complete health, as he then considered himself, he dropped
down in the street and expired."
XXL On the Discrimination of Chronic Rheumatism, from
Gout, Acute Rheumatism) Scrophala, Nodosity, White
Swelling, and other painful Diseases of the Joints and
Muscles. By John Haygarth, M.D. F.R.S. and F.R.S.
Edinburgh.
This is an excellent dissertation upon a class of diseases
to which the author has long devoted peculiar attention.
XXII. On the Climacteric Disease. By Sir H. Hal-
ford, Bart. F.R.S.
In this essay, the learned baronet has presented us with
an interesting account of'what we have hitherto regarded
merely as a general decay of the system in the latter period
of life, but which he is disposed to consider as ? disease,
which he describes " as a falling away of the flesh in the
decline of life, without any obvious source of exhaustion,
accompanied with a quicker pulse than natural, and an ex-
traordinary alteration in the expression of the countenance.
" Sometimes the disorder comes on so gradually and in-
sensibly, that the patient is hardly aware of its commence-
ment. He perceives that he is sooner tired than usual, and
that he is thinner than he was ; but yet he has nothing ma-
terial to complain of. In process of time his appetite be-
comes seriously impaired; his nights are sleepless, or if he
get sleep, he is not refreshed by it. His face becomes visibly
extenuated, or perhaps acquires a bloated look. His tongue
is white, and he suspects that he has fever.
" If he ask advice, his pulse is found quicker than fc
should be, and he acknowledges that he has felt pains occa-
sionally in his head and chest, and that his legs are disposed
to swell j yet there is no deficiency in the quantity of his
y 2 iirine3
154 Transactions of the College of Physicians.
urine, nor any other sensible failure in the action of the
abdominal viscera, excepting that the bowels are more
sluggish than they used to be.
" Sometimes the head-ach is accompanied with vertigo;
and sometimes severe rheumatic pains, as the patient believes
them to be, are felt in various parts of the body, and in the
limbs ; but, on inquiry, these have not the ordinary seat, nor
the common accompaniments of rheumatism, and seem
rather to take the course of the nerves, than of the muscular
fibres.
" In the latter stages of this disease, the stomach seems
to lose all its powers; the frame becomes more and more
emaciated; the cellular membrane, in the lower limbs, is
laden with fluid ; there is an insurmountable restlessness by
day, and a total Want of sleep at night; the mind grows
torpid and indifferent to what formerly interested it; and
the patient sinks at last, seeming rather to cease to live, than
to die of a mortal distemper.
" Such is the ordinary course of this disorder in its most
simple form, when it proves fatal. When the powers of the
constitution are superior to the influence of the malady, the
patient loses his symptoms gradually, recovers his rest and
his appetite, and, to a certain degree, his muscular strength
and flesh; but the energies of his frame are never again
?what they were before, nor does the countenance recover
its former volume and expression."
Sir Henry remarks, that the disease is seldom seen in its
simple fcrm, it being generally complicated with other com-
plaints and assuming their character, and accompanying
them in their course. The most frequent cause of it is stated
to be a common cold: it is sometimes the result of an act of
intemperance; of a fall; and of a marriage contracted late in
life; anxiety and sorrow. The author avoids encouraging
us to hope much from medicine in the cure of this affection,
and only is confident in cautioning practitioners against re-
sorting to too active a treatment.
XXIII. On Abdominal Tumor, originating in Lumbar
Abscess? By J. Latham, M.D. F.R.S.
Dr. Latham suspects that he has seen three cases where
matter originally formed in the loins has been collected into
a chronic abscess within the abdomen, and made a passage
for itself through the vagina.
XXIV. A Case of Intestinal Protrusion per A num. Com-
municated by Henry Dampier, Esq. Barrister at Law, to
John Latham, M.D.
Although this extraordinary case has passed the College
ordeal,
Dr. Latham on Intestinal Protrusion per j num. 165
ordeal, we suspect that some of our brethren, less gifted
with faith, will doubt its possibility. Dr. Latham, in his
introduction to it, says, " he is not aware of any similar
instance having occurred." We copy it for the edification
of our readers.
" On the 3d of January, 1735, I was called to John, the
son of Lancelot Watts (a day-laborer) living at Brunsted, a
servant boy to Mr. Pile, a farmer at Westwick, near North
Walsham, Norfolk, aged thirteen years: he was overturned
in a cart, and thrown flat on his face, with the round or
edge of one side of the cart (bottom upwards) whelmed
across his loins; the upper part of the body lying beyond
the wheel at right angles. In this helpless condition he con-
tinued some time, and-was found with a very large portion
of the intestines forced out at the anus, with the mesentery,
and some loose pieces of fat, which t took to be part of the
omentum hanging down below the hams, double, like the
reins of a bridle, very much distended and inflamed ; he had
a continual nausea, and violent retchings to vomit, and
threw up every thing he took ; the pain Of the stomach and
bowels was exquisite, attended with convulsions, pulse low
and quick, and frequently he fell into cold sweats: after
using an emollient and spirituous fomentation, I reduced
the parts, though.to no purpose; the vomiting immediately
returned and forced them out again. Next day the fever
increased; the nausea and retchings to vomit continued j
the parts appeared livid and black, with all signs of a morti-
fication. On the third day the mortification increasing, I
cut off the intestine, with the mesentery, close to the anus.
He had had no stool from the time of the accident; but soon
after the operation there was a very large discharge of
blackish and extremely offensive faices, which continued
several days, lessening by degrees. He soon grew easy;
the nausea and vomiting abated. I gave him Tinct. cort.
Pernv. simpl. twice a-day; and as he complained at times of
griping pains, he took now and then Tinct. Rhatan. vinos
and has recovered a good state of health.
" For some time he had six or seven or more stools in a
daj-; at present commonly three or four, all loose, which
come soon after eating, and frequently he is obliged to hurry
out to ease himself during his meals.
ii 1 have three times lately tried if I could discover a
passage through the coats of the rectum with my finger, and
I think 1 have always felt an opening just above the sphinc-
ter, towards the spine, the circumference of which was.full,
and protuberated seemingly as large as my finger, the lower
edge
16G Transactions of the College of Physicians.
edge of which was harder than the rest. He complained of
pain when I pressed the upper part.
" The intestine cut oil' measured fifty-seven inches by a
string applied to the outer surface.
" On the 7th of May, the boy came walking from Brunsted
to North Walsham (seven miles), and dined with me ; was
perfectly well, and walked back again that afternoon."
The Rev. Mr. Dampier, in a memorandum at the end of
the case, states that this account was given him at Mr. Wind-
ham's, at Felbrigg in Norfolk, by his apothecary, Mr. Need-
bam, of North Walsham, who likewise showed him the
above-mentioned intestine, which was cut off and preserved
in spirits.?Dated, December 175(5.
XXV. Case of Hydrophobia. By Richard Patrick Sat-
terley, M. D.
This is another fatal instance of Hydrophobia. The pa-
tient was a girl aged eight years; and was received in the
Middlesex Hospital two days after the symptoms commenced.
She of course had all the attention and able assistance which
her horrible complaint would naturally claim. The case is
minutely related, but we can only afford space for the
author's account of the dissection.
" Fourteen hours after death the bod}? was opened by my
friend Mr. Cartwright, one of the surgeons of the hospital.
" The bitten part was cut through in various directions,
but no trace of diseased action could be discovered in it.
The salivary glands, and the glands of the neck, were in a
sound state. The trachea was nearly filled with a thick and
glutinous mucus, which was continued into the minute rami-
fications of the bronchia?, with this trifling difference, that
the mucus in the lungs was somewhat thinner. There were
patches of inflammation upon the internal surface of the
trachea, more particularly on its posterior part.
" The oesophagus, from the part where it dips into the
chest, and for about three inches towards the stomach, was
lined with a thin semi-transparent layer of coagulable lymph.
The internal surface of the stomach had nothing unusual in
its appearance, except at the small arch, where, in a circle
of about five inches diameter, it was of a dark bloody hue,
approaching to black. At first this appearance resembled
the effusion of blood under the villous coat, but it was found
to be an exudation from the extremities of the blood-vessels
upon the surface of the stomach, capable of being renewed
in a tew minutes after it had been eleaned away by a wet
sponge. No abrasion of the villous coat was discoverable,
nor were any clots, of blood mixed with the contents of the
3 stomach.
stomach. Neither the upper orifice nor the pylorus, nor
the intestinal canal, showed marks of disease. The brain
was in a sound state, unless we except a turgescency of th&
vessels of the pia mater" '
The remaining article in this volume contains the Report
of the College on Vaccination, which has already appeared
in the Medical Journal. Our extracts have been large, he-
cause we presumed they would be acceptable to many of
our country connections, who might not have an opportunity
of obtaining the volume itself, tne most valuable portion of
which they may thus possess. We are also happy to an-
nounce that a fifth volume is expected shortly.

				

